# Supplementary material for: Automated mold defects classification in paintings: A comparison of machine learning and rule-based techniques
Source: PLoS One. 2025 Jan 24;20(1):e0316996. doi: 10.1371/journal.pone.0316996 (PMC11761149; doi:10.1371/journal.pone.0316996)
Supplement: Attachment — Submitted filename: Response to Reviewers.docx [file pone.0316996.s001.docx]

11^th^ November 2024

Yile Chen, Ph.D. in Architecture
Academic Editor
*PLOS ONE*

Dear Editor:

We are writing to thank you and the reviewers for their thoughtful comments on our manuscript, “Automated Mold Defect Classification in Paintings: A Comparison of Machine Learning and Rule-Based Techniques”. We have carefully considered their suggestions and believe that our revised manuscript significantly addresses the points raised. Here are the response to each of the points raised by the reviewers:

**Reviewer 1 Comments**

- Comment 1: In Introduction, the last paragraph citations must be revised carefully.
- Response 1: Thank you for the feedback. The last paragraph in the introduction containing the citations has been revised for clarity (**see line 73**). A concluding remark has also been added at the end of the paragraph.

- Comment 2: In Related Works, I expected to see a comparison between previous models (techniques) according to advantages and disadvantages as minimum.
- Response 2: Thank you for the feedback. Two paragraphs were added to explain the advantages and disadvantages of the techniques discussed in the paper (**see line 102**).
- Comment 3: In Methodology, the first paragraph shouldn't be about the dataset. But it should be about the proposed model.
- Response 3: Thank you for the feedback. The first paragraph has been revised to explain about the proposed model rather than the dataset (**see line 130**).
- Comment 4: In Methodology, Table 1 caption should be rephrased.
- Response 4: Thank you for your feedback. We have reviewed the caption for Table 1 and made the necessary revisions. The updated caption now reads (**see line 172**):
  - “Distribution of Quantitative Mold Features used in the Classification Models”
- Comment 5: In Results and Discussion, Table 2 must be sorted according to the most important evaluation metric/s.
- Response 5: Thank you for your valuable feedback. We have revised Table 2 to sort results according to the most important evaluation metric, Detection. This provides a clearer and a more focused comparison of the different methods (**see line 287**).
- Comment 6: A comparison between previous and proposed techniques should be considered in Results and Discussion section.
- Response 6: We have carefully considered your suggestions and incorporated significance analysis to compare the previous and the proposed techniques in the Results and Discussion section (**see line 309**).
- Comment 7: The small size of the dataset used may affect the accuracy of models. This must be considered in the future work.
- Response 7: We appreciate the reviewer’s comment regarding the potential impact of dataset size on model accuracy. We acknowledge that a larger dataset could enhance our results. In future work, we plan to apply data augmentation techniques, explore the use of Convolutional Neural Network, using multiple feature extraction algorithms and addressing the issue of dataset class imbalance (**see line 354**).

**Reviewer 2 Comments**

- Comment 1: The statistical analysis, while generally sound, could be more rigorous. The authors should consider adding significance tests to compare the performance of the different methods and providing confidence intervals for the performance estimates. Hyperparameter tuning for the machine learning models could also be explored
- Response 1:
  - We agree that strengthening the statistical analysis would enhance the robustness of our findings. We have incorporated the use of significance test in the form of paired t-test and reported the p-values to assess the significance of it in Table 3. We also have added another literature to the references to support the approach (**see line 309**).
  - While we acknowledge that hyperparameter tuning can potentially improve model performance, we chose to use the machine learning model as it is to establish baseline comparison with rule-based method. In future studies, we plan to explore hyperparameter tuning to optimize our models and potentially achieve better results (**see line 366**).
- Comment 2: There are some minor typographical and grammatical errors throughout the manuscript that should be corrected in a revision.
- Response 2: The manuscript has been sent for proofreading and typographical and grammatical errors throughout the manuscript has been addressed.

Please address all correspondence concerning this manuscript to me at norrimamokhtar@um.edu.my

Thank you for your time and consideration of our manuscript.

Sincerely,

Assoc. Prof. Ir. Dr. Norrima binti Mokhtar
Department of Electrical Engineering,
Faculty of Engineering,
Universiti Malaya
